# Cecal microbiota transplantation: unique influence of cecal microbiota from divergently selected inbred donor lines on cecal microbial profile, serotonergic activity, and aggressive behavior of recipient chickens

**DOI:** 10.1186/s40104-023-00866-9

**Published:** 2023-05-02

**Authors:** Yuechi Fu, Jiaying Hu, Marisa A. Erasmus, Huanmin Zhang, Timothy A. Johnson, Hengwei Cheng

**Affiliations:** 1 Department of Animal Sciences, Purdue University, West Lafayette, IN 47907 USA; 2 Avian Disease and Oncology Laboratory, USDA-ARS, East Lansing, MI 48823 USA; 3 Livestock Behavior Research Unit, USDA-ARS, West Lafayette, IN 47907 USA

**Keywords:** Aggressive behavior, Cecal microbiota transplantation, Chicken, Microbiota-gut-brain axis, Serotonergic activity

## Abstract

**Background:**

Accumulating evidence from human trials and rodent studies has indicated that modulation of gut microbiota affects host physiological homeostasis and behavioral characteristics. Similarly, alterations in gut microbiota could be a feasible strategy for reducing aggressive behavior and improving health in chickens. The study was conducted to determine the effects of early-life cecal microbiota transplantation (CMT) on cecal microbial composition, brain serotonergic activity, and aggressive behavior of recipient chickens.

**Methods:**

Chicken lines 6_3_ and 7_2_ with nonaggressive and aggressive behavior, respectively, were used as donors and a commercial strain Dekalb XL was used as recipients for CMT. Eighty-four 1-d-old male chicks were randomly assigned to 1 of 3 treatments with 7 cages per treatment and 4 chickens per cage (*n* = 7): saline (control, CTRL), cecal solution of line 6_3_ (6_3_-CMT), and cecal solution of line 7_2_ (7_2_-CMT). Transplantation was conducted via oral gavage once daily from d 1 to 10, and then boosted once weekly from week 3 to 5. At weeks 5 and 16, home-cage behavior was recorded, and chickens with similar body weights were assigned to paired aggression tests between the treatments. Samples of blood, brain, and cecal content were collected from the post-tested chickens to detect CMT-induced biological and microbiota changes.

**Results:**

6_3_-CMT chickens displayed less aggressive behavior with a higher hypothalamic serotonergic activity at week 5. Correspondingly, two amplicon sequence variants (ASVs) belonging to Lachnospiraceae and one *Ruminococcaceae UCG-005* ASV were positively correlated with the levels of brain tryptophan and serotonin, respectively. 7_2_-CMT chickens had lower levels of brain norepinephrine and dopamine at week 5 with higher levels of plasma serotonin and tryptophan at week 16. ASVs belonging to Mollicutes RF39 and *GCA-900066225* in 7_2_-CMT chickens were negatively correlated with the brain 5-hydroxyindoleacetic acid (5-HIAA) at week 5, and one *Bacteroides* ASV was negatively correlated with plasma serotonin at week 16.

**Conclusion:**

Results indicate that CMT at an early age could regulate aggressive behavior via modulating the cecal microbial composition, together with central serotonergic and catecholaminergic systems in recipient chickens. The selected CMT could be a novel strategy for reducing aggressive behavior through regulating signaling along the microbiota-gut-brain axis.

## Introduction

Aggression in psychology refers to a range of behaviors resulting in physical, physiological, and psychological harm within a social group. As an evolutionarily conserved behavior, aggression exists widely across the animal kingdom from humans to various animals including chickens. From the evolutionary perspective, aggression serves as a crucial skill for survival and reproduction enabling an animal to secure resources and adapt to the surrounding environment [1]. In chickens, the origin of aggression can be traced back to their wild ancestors (red junglefowl *Gallus gallus*) who naturally live within a small group that maintains a dominance hierarchy by showing establishment fights towards individuals, with the memory and recognition of past encounters (pecking order or social rank) through visual cues such as body size, head, appendages, and comb [2, 3]. However, based on the cost–benefit analysis, aggression and related damaging behaviors, such as violence within a group, cause social stress, interfere with societal safety, and damage the health of both dominants and subordinates [4, 5]. In the modern intensive animal production system, some routine management practices, such as mixing and regrouping unfamiliar animals, may disrupt social structural stability and increase aggressive behavior among individuals, compromising animal production, health, and welfare [6]. In chickens, aggression leads to excessive stress, injuries, and even death [7], which results in huge economic losses for the poultry industry. Thus, it is pivotal to develop an approach to control aggression, thereby improving bird health and increasing the economic profits of the poultry industry.

In recent years, many studies have focused on the gut microbiota and their link with host physiological and psychological health via the functional regulation of the gut-brain axis. The first evidence for the existence of the gut-brain axis was reported in the early nineteenth century when researchers found that a person’s emotional state could govern gut health [8]. Correspondingly, the gut microbiota functions as an endocrine organ, influencing both mood stabilization and behavioral exhibition of the host via regulating the biofunctions of neurotransmitters and neurotrophic factors [9], immune process [10], and energy metabolism [11]. In humans, perturbation of the gut microbiota composition has been linked to the pathophysiology of mental disorders [12]. For instance, patients with depression have increased abundances of Bacteroidetes and Proteobacteria as compared to healthy individuals [13]. Microbiota transplantation studies in germ-free (GF) mice have further revealed the causal role of gut microbiota in regulating brain functions. For example, fecal microbiota transplantation (FMT) from patients with depression induces the specific depression-like behavior, together with a decrease in several brain neurotransmitters such as serotonin (5-HT), norepinephrine (NE), and epinephrine (EP) in GF recipients [14]. Similar findings of FMT eliciting the phenotype of the donors in the recipients have been evidenced in several psychiatric disorders including autism [15], schizophrenia [16], and Alzheimer’s disease [17], which further indicates the functional link between the gut microbiota and the brain, i.e., the microbiota-gut-brain axis. Based on these findings, we hypothesized that the gut microbiota may present a similar function in regulating aggressive behavior in chickens.

The regulation of aggressive behavior is primarily mediated through altering central neuronal activities [18]. Based on the 5-HT deficiency hypothesis of aggression [19], 5-HT is a primary modulatory neurotransmitter involved in this process. In humans, diminished serotonergic function within the central nervous system (CNS) has been linked with mood and behavioral disorders including interpersonal aggression and impulsive behavior [20]. Similarly, aggressive chickens usually exhibit lower 5-HT concentrations in specific brain regions, such as the hypothalamus [21]. Also, it has been reported that dietary supplementation with tryptophan (TRP), the precursor of 5-HT, reduces fear in quail [22], aggression in broilers [23], and teleost fishes [24].

Highly inbred chicken lines 6_3_ and 7_2_ have been continuously selected for resistance and susceptibility, respectively, to Marek’s disease for more than 60 years [25, 26]. It has also been evidenced line’s difference in neuroendocrine systems, immunity [27, 28], gut microbiome composition [29], and aggressive behavior [30]. In response to social stress, line 7_2_ chickens are more aggressive than line 6_3_ chickens. Consistent with these findings, line 7_2_ roosters exhibit lower levels of brain 5-HT than line 6_3_ roosters [31]. Considering the essential role of brain serotonergic mediation in aggression, the objective of this study was to determine if early-life cecal microbiota transplantation (CMT) from the divergently selected donors (lines 6_3_ and 7_2_) would potentially reduce aggressive behaviors in recipients through modulating the cecal microbial composition and the central serotonergic system via the gut-brain axis.

## Methods

### Study design and treatment

**Fig. 1:**
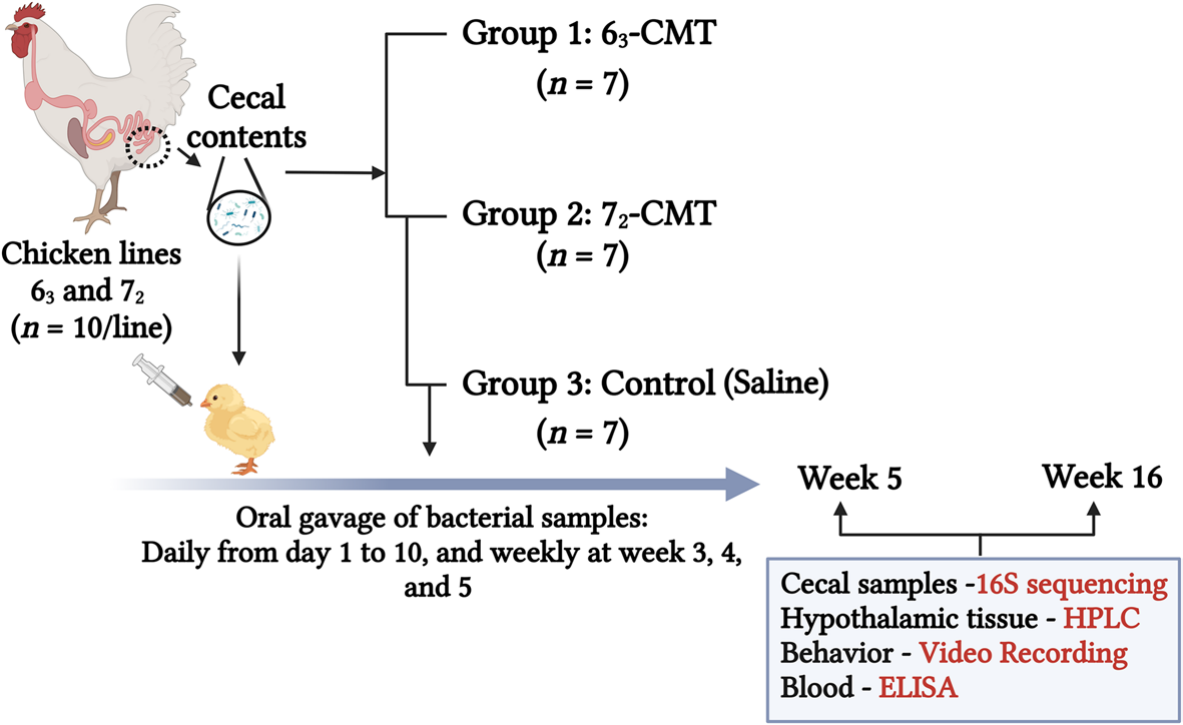
Schematic view of the experimental design (created with BioRender.com.). 6_3_-CMT, received cecal content solution from 6_3_ donors; 7_2_-CMT, received cecal content solution from 7_2_ donors; CTRL, received saline, control

The experimental workflow is summarized in Fig. 1. Inbred chicken lines 6_3_ and 7_2_ selected for Marek’s disease resistance or susceptibility (the Avian Disease and Oncology Laboratory, East Lansing, MI, USA) were used as donors [25]. Cecal contents were randomly collected from 10 sixty-week-old hens per line after 60 generations of selection and then evenly pooled within the line. Five grams of pooled cecal contents were suspended with gut microbiome media at a ratio of 1:10, then kept at −20 °C until oral gavage [32]. A total of 84 day-old Dekalb XL chicks, a commercial strain, was used as recipients and randomly assigned to 1 of 3 treatments with 7 cages per treatment and 4 chickens per cage (*n* = 7): CTRL (0.1 mL saline, control), 6_3_-CMT (0.1 mL of line 6_3_ cecal content solution), and 7_2_-CMT (0.1 mL of line 7_2_ cecal content solution) for a 16-week trial. Oral gavage was performed daily from d 1 to 10, and then boosted weekly from week 3 to 5. Samples of blood, hypothalamus, and both sides of cecal contents were collected from 1 bird per cage at week 5 (5 d after the boost) and week 16, respectively. A 5-mL blood sample was collected through the brachial vein of each sampled bird using an EDTA-coated tube. The blood samples were centrifuged at 700 × *g* for 15 min at 4 °C, and then plasma was separated and stored at −80 °C until further analysis. The sampled chickens were then killed by cervical dislocation for tissue collection (i.e., cecal contents and hypothalamic tissues). The hypothalamus was dissected from each sampled bird according to brain anatomical landmarks described previously [33, 34]. After collection, cecal contents and hypothalamic tissues were flash frozen and stored at −80 °C until analysis. Water and feed were provided ad libitum. The general management, including vaccination, dietary formulation and nutrient contents, ambient temperature and humidity, and lighting program was followed the Dekalb White management guideline [35].

### Behavioral analysis

#### Home-cage aggressive behavior

The behavior of chickens in the home cage was recorded for a 24-h period at week 5 and 16 (*n* = 5) using digital video recording (DVR) systems (Geovision GV-1480) linked to overhead cameras (Panasonic WV-CP254H). The recorded video was viewed using EZViewLog500 in real-time and collected from 1 h after lights on (at 5:58) and 1 h before lights off (at 19:58). The aggressive pecking was determined following the definition described previously: “*Occurs when one bird raises head and forcefully stabs beak either once or multiple times at the head or body of another bird. The recipient will usually show avoidance behavior by ducking or moving away from aggressive chickens”* [36]. The frequency of aggressive pecking was counted during 2-h video segments and calculated as the average number of pecks per bird per hour (the number of pecks per cage per hour = total number of pecks/2 h/the number of chickens in each cage).

#### Paired behavioral test

At week 5 and 16, paired behavioral tests were conducted between the treatments following the procedure published previously [37]. Briefly, chickens with similar BW among the treatments were randomly assigned to pairs for aggression tests in novel cages (*n* = 7) and the body weight of chickens at each test time was shown in Table 1. All tests were conducted during the same period in the morning between 8:00 to 24:00 in the test room and each pair was continuously recorded for 30 min. Afterward, the frequency of aggressive behavior was counted and calculated as the average number of aggressive pecks per bird per pair for statistical analysis.

**Table 1:** Body weight of pair-tested birds at week 5 and week 16

Treatment		Body weight/g	SEM	*P*-value
Week 5				
6_3_-CMT vs. 7_2_-CMT	6_3_-CMT	374	17.93	0.78
	7_2_-CMT	381	14.97	
6_3_-CMT vs. CTRL	6_3_-CMT	354	15.28	0.81
	CTRL	360	18.35	
7_2_-CMT vs. CTRL	7_2_-CMT	413	9.93	0.42
	CTRL	406	10.82	
Week 16				
6_3_-CMT vs. 7_2_-CMT	6_3_-CMT	1694	27.24	0.62
	7_2_-CMT	1733	23.47	
6_3_-CMT vs. CTRL	6_3_-CMT	1762	26.09	0.62
	CTRL	1721	22.32	
7_2_-CMT vs. CTRL	7_2_-CMT	1806	19.41	0.56
	CTRL	1789	24.76	

*6*_*3*_*-CMT* Received cecal content solution from 6_3_ donors, *7*_*2*_*-CMT* Received cecal content solution from 7_2_ donors, *CTRL* Received saline, control

### Cecal microbial profiles

#### 16S rRNA amplicon sequencing

Total genomic DNA was extracted from the cecal samples using the QIAamp Fast DNA Stool Mini kit (Catalog #: 51504, Qiagen, Valencia, CA, USA) following the manufacturer’s instructions. The 16S rRNA gene sequence was sequenced and analyzed to characterize the effects of CMT on the gut microbial community diversity and structures of the recipient chickens [38]. Briefly, the V4 region of the 16S rRNA genes was amplified from the genomic DNA using 515F (5'-GTGCCAGCMGCCGCGGTAA-3') and 806R (5'-GGACTACHVGGGTWTCTAAT-3'). Each 25 µL PCR reaction consisted of 9.5 µL of PCR-grade water, 12.5 µL of QuantaBio’s AccuStart II PCR ToughMix (2 × concentration, 1 × final), 1 µL Golay barcode tagged Forward Primer (5 µmol/L, 200 pmol/L final), 1 µL Reverse Primer (5 µmol/L, 200 pmol/L final), and 1 µL of template DNA. The conditions for PCR were as follows: 94 °C for 3 min to denature the DNA, 35 cycles at 94 °C for 45 s, 50 °C for 60 s, and 72 °C for 90 s; then 10 min at 72 °C to ensure complete amplification. The amplicons were quantified using the PicoGreen assay (Invitrogen, Carlsbad, CA, USA). Afterward, the volume were pooled into a single tube and cleaned up using the AMPure XP Beads (Beckman Coulter, Brea, CA, USA), then quantified using a fluorometer (Qubit, Invitrogen, Carlsbad, CA, USA). After quantification, the molarity of the pool was determined and diluted down to 2 nmol/L, denatured, then diluted to a final concentration of 6.75 pmol/L with a 10% PhiX spike [39]. The amplicons were sequenced on an Illumina Miseq platform using 2 × 150 bp paired-end reads at the Argonne National Laboratory (Chicago, IL, USA).

#### Sequence analysis

The amplicons were trimmed at the 13^th^ base of each sequence for both the forward and reversed sequences and denoised with the Divisive Amplicon Denoising Algorithm (DADA 2) pipelines via the Quantitative Insights into Microbial Ecology (QIIME, v2020.2). The DADA generated amplicon sequence variants (ASVs) were classified using the 99% Silva v132 taxonomic database as a reference (https://data.qiime2.org/2020.2/common/silva-132-99-515-806-nb-classifier). Cecal samples were rarefied to 13,580 and 24,907 reads at week 5 and 16, respectively. The α-diversity was evaluated by the Faith’s PD index. To visualize the dissimilarity of microbial communities, the plots of principal coordinate analysis (PCoA) based on unweighted UniFrac were performed using the Phyloseq, DESeq2, and ggplot2 packages in addition to custom scripts in the R studio (version 3.6.2). Treatment effects were evaluated using the Adonis, and the pairwise comparison was assessed using the permutational multivariate analysis of variance (PERMANOVA) test. The false discovery rate (FDR) adjusted *P*-value (*q*-value) was used for multiple comparisons with the QIIME 2. The R studio (version 3.6.2) was performed to determine the treatment effect on the differential abundant ASV at the phylum and genus levels using the DESeq2 package (version 1.30.1). Significant differences in log_2_ fold changes were determined by the Wald test with *P*-value correction using the Benjamin and Hochberg test. Levels of significance were determined when *P* < 0.05 and LDA score > 2.

### Brain serotonergic activities

The examined genes involved in the serotonergic pathways were listed in Table 2. Total RNA of the hypothalamic samples was extracted using the RNeasy Mini Lipid Kit (Catalog #: 74804, Qiagen, Valencia, CA, USA) following the instructions provided by the company. The purity and concentration of total RNA were checked using a NanoDrop 2000 (Thermo Scientific, Wilmington, DE, USA). The reverse transcription was conducted by the Reverse Transcription Reagent Pack (Catalog #: N8080234, Applied Biosystems, Foster City, CA, USA). A mixture of reverse transcription reagents consisted of 2 μL RNase inhibitor, 2.5 μL multi-scribe reverse transcriptase, 5 μL random hexamers, 10 μL of TaqMan reverse transcription buffer, 20 μL deoxy nucleotides, and 22 μL of magnesium chloride. A total mixture of each sample consisted of 61.5 μL with the adjusted volume of RNA sample adjusted volume of RNA sample and RNase-free water for a final 100 μL. The RNA samples were reverse transcribed to cDNA using a Techne TC-3000G PCR Thermal Cycler (Bibby Scientific Limited, Stone, UK). To explore the effects of CMT on the activities of serotonin pathway, mRNA abundance of related genes (Table 2) in the left side of hypothalamic tissues were detected by RT-qPCR using the protocol described previously [40]. Glyceraldehyde 3-phosphate dehydrogenase (*GAPDH*) was used as a housekeeping gene. The cycling conditions were 50 °C for 2 min and 95 °C for 10 min of the holding stage, followed by 40 cycles of 95 °C for 15 s, then 60 °C for 1 min. Results were quantitated using the standard curve method. The standards were measured in triplicates with a standard deviation of less than 2.0 and a coefficient of variation less than 2.0%.

**Table 2:** Relevant genes involved in the central serotonergic pathway

Function	Genes	Assay ID
Synthesis	*TPH2*	Gg03345550_m1
Reuptake	*5-HTT*	Gg03349687_m1
Auto-receptor signaling	*Htr1a*, *Htr1b*	Gg07157052_s1, Gg07157117_s1
Metabolism	*MAOA*	Gg03350714_m1

### Brain monoamine profiles

To determine the effects of CMT on central serotonergic activities of recipient chickens, duplicate samples were run by HPLC (UltiMate™ 3000 RSLCnano System, Thermo Fisher Scientific Inc., Waltham, MA, USA) as previously described [41]. Briefly, the right side of the hypothalamus was weighed and homogenized in 0.2 mol/L perchloric acid at a 10:1 ratio and then vortexed for 1 min. Afterward, the supernatant was centrifuged at 14,000 r/min for 10 min at 4 °C. The supernatants were drawn into a microcentrifuge tube and diluted with MD-TM mobile phase (Thermo Fisher Scientific Inc., Waltham, MA, USA) at 1:1. The flow rate of the mobile phase was 0.8 mL/min. Duplicate samples were run at 10 μL per injection. The concentrations of 5-hydroxyindoleacetic acid (5-HIAA), serotonin (5-HT), tryptophan (TRP), norepinephrine (NE), epinephrine (EP), and dopamine (DA) were calculated as nanograms per gram of wet tissue (ng/g) through a reference curve generated from the corresponding standards.

### Peripheral serotonin and tryptophan

The concentrations of 5-HT and TRP (My BioSource, San Diego, CA, USA) in the plasma were measured using the respective ELISA kits following the relative company’s instructions. Briefly, 50 μL of samples were incubated with 100 μL of horseradish peroxidase (HRP) conjugate reagent at 37 °C for 60 min. Afterward, the plates were washed and then 50 μL of chromogen solutions A and B were added for ELISA detection at 37 °C for 15 min. Thereafter, stop solution (0.25 mol/L H_2_SO_4_) were added and the absorbance was monitored at 450 nm within 5 min.

### Statistical analysis

The fixed effects were treatment and age. Physiological and behavioral data were considered as response variables. Data were analyzed using R studio (version 3.6.2) except for home-cage behavioral data, which were analyzed using PROC MIXED repeated measures procedures of SAS 9.4 (SAS Institute Inc., Cary, NC, USA). The frequency of aggressive pecking in home cages was normalized using Box-Cox transformation before analysis. The behavioral data of the paired test were analyzed using the Kruskal–Wallis test and followed by Dunn’s test for multiple comparisons. Other normalized physiological data was carried out by one-way ANOVA and the Tukey–Kramer test was used to partition any significant differences among the least-square means due to the treatment effect [42]. For each treatment, Spearman’s rank test was used to find the correlation between the significantly differential abundant ASVs and differential neurotransmitter concentrations. Significance was set at *P* ≤ 0.05 and a trend was defined as 0.05 < *P* ≤ 0.1.

## Results

### Differences in aggressive behavior of recipient chickens

**Fig. 2:**
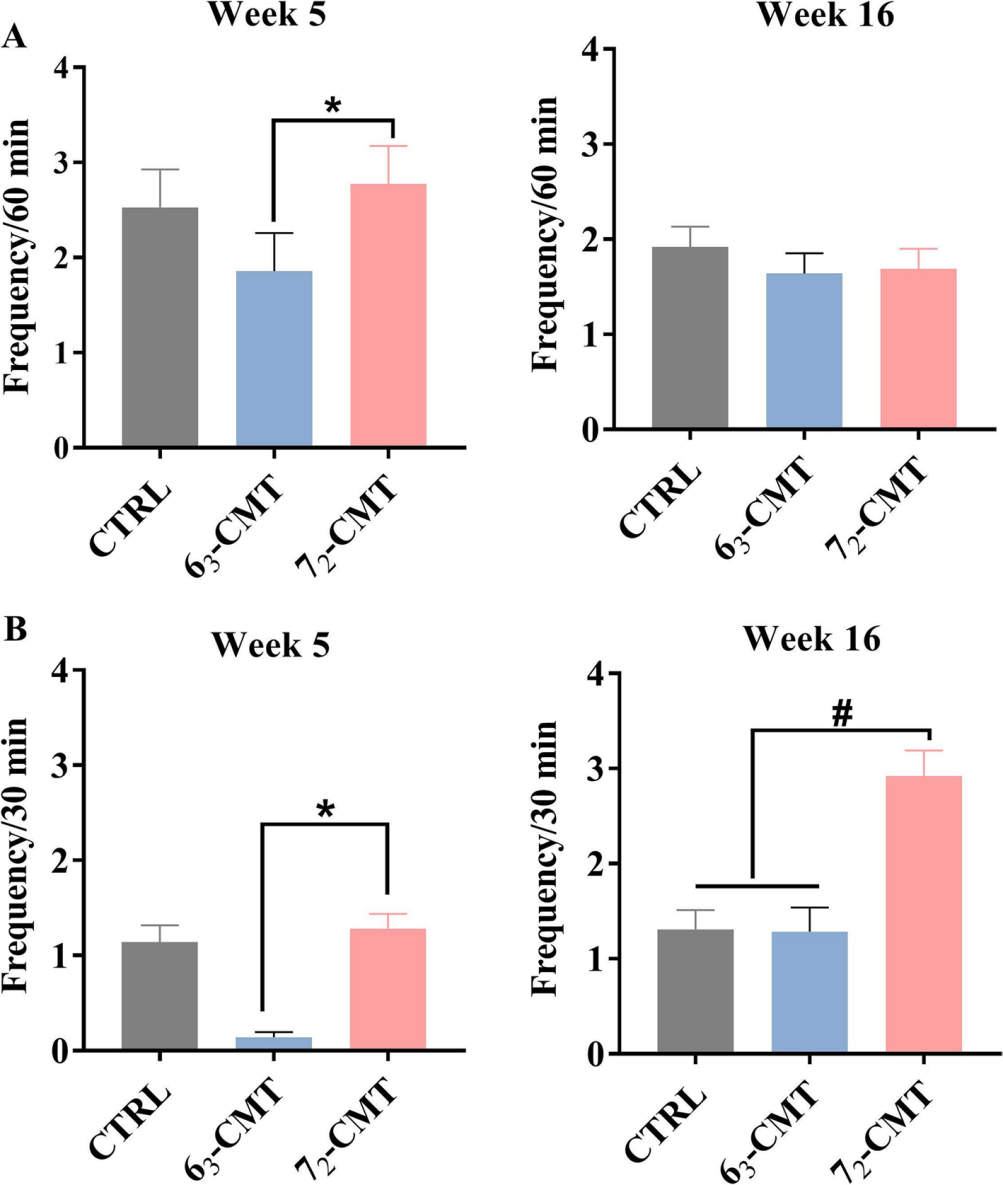
Frequency of aggressive pecking of recipient chickens at week 5 and week 16. **A** Home-cage behavior **B** Paired test. Values are means ± SEM, *n* = 7. *Indicates significant differences (*P* ≤ 0.05), and ^#^shows trend differences (0.05 < *P* ≤ 0.1). 6_3_-CMT, received cecal content solution from 6_3_ donors; 7_2_-CMT, received cecal content solution from 7_2_ donors; CTRL, received saline, control

Compared to 7_2_-CMT chickens, 6_3_-CMT chickens displayed less aggressive pecking in home cages (*P* = 0.039, Fig. 2A) and paired tests at week 5 (*P* = 0.040, Fig. 2B). At week 16, 7_2_-CMT chickens tended to exhibit more aggressive pecking than both 6_3_-CMT and CTRL chickens during paired tests (*P* = 0.093) but not in home cages (*P* = 0.455).

### Changes of the cecal microbial profiles in recipient chickens

**Fig. 3:**
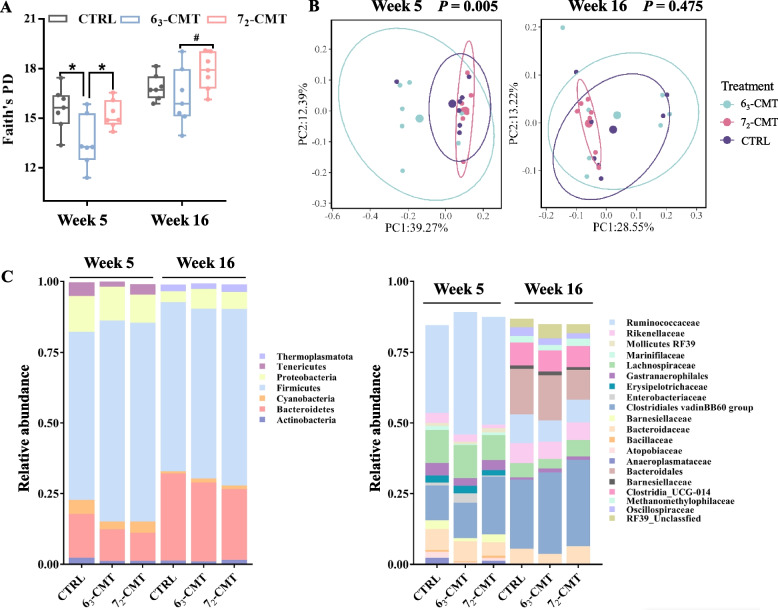
Effects of cecal microbiota transplantation on cecal microbial profiles of recipient chickens at week 5 and week 16 (*n* = 7). **A** Faith’s PD index, values are median ± SEM, ^*^Indicates significant differences (*P* ≤ 0.05), and ^#^ shows trend differences (0.05 < *P* ≤ 0.1). **B** Principal coordinate analysis (PCoA) of Unweighted UniFrac of recipient chickens at week 5 and week 16. Each dot represents one bird (*n* = 7), PCo1 and PCo2 represent the percentage of variance explained by each coordinate. **C** Cecal microbial composition profiles of the recipient chickens at phylum and family **(**relative abundance > 1%) levels. 6_3_-CMT, received cecal content solution from 6_3_ donors; 7_2_-CMT, received cecal content solution from 7_2_ donors; CTRL, received saline, control

Cecal microbiota transplantation induced different effects on the cecal microbial structure and diversity of the recipient chickens (Table 3 and Fig. 3). At week 5, 6_3_-CMT chickens had the lowest phylogenetic diversity (Faith’s PD index) among the treatments (*P* = 0.033, Fig. 3A), and tended to be lower than 7_2_-CMT chickens at week 16 (*P* = 0.090). Also, treatment effects on Unweighted UniFrac were found in the recipient chickens at week 5 (*P* = 0.005) but not at week 16 (*P* > 0.05, Fig. 3B). The post-hoc pairwise test showed that there were significant differences in the microbial community structures between 6_3_-CMT chickens and 7_2_-CMT chickens (*P* = 0.006, Table 3). Community composition at phylum and family levels are shown in Fig. 3C.

**Table 3:** The effects of cecal microbiota transplantation on Unweighted_UniFrac metric in recipient chickens at week 5

Pairwise PERMANOVA					
Test between treatments		Permutations	pseudo-F	*P*-value	*q*-value
CTRL	7_2_-CMT	999	1.605	0.036	**0.036**
CTRL	6_3_-CMT	999	2.751	0.036	**0.036**
7_2_-CMT	6_3_-CMT	999	4.587	0.002	**0.006**

*6*_*3*_*-CMT* Received cecal content solution from 6_3_ donors, *7*_*2*_*-CMT* Received cecal content solution from 7_2_ donors, *CTRL* Received saline, control

**Fig. 4:**
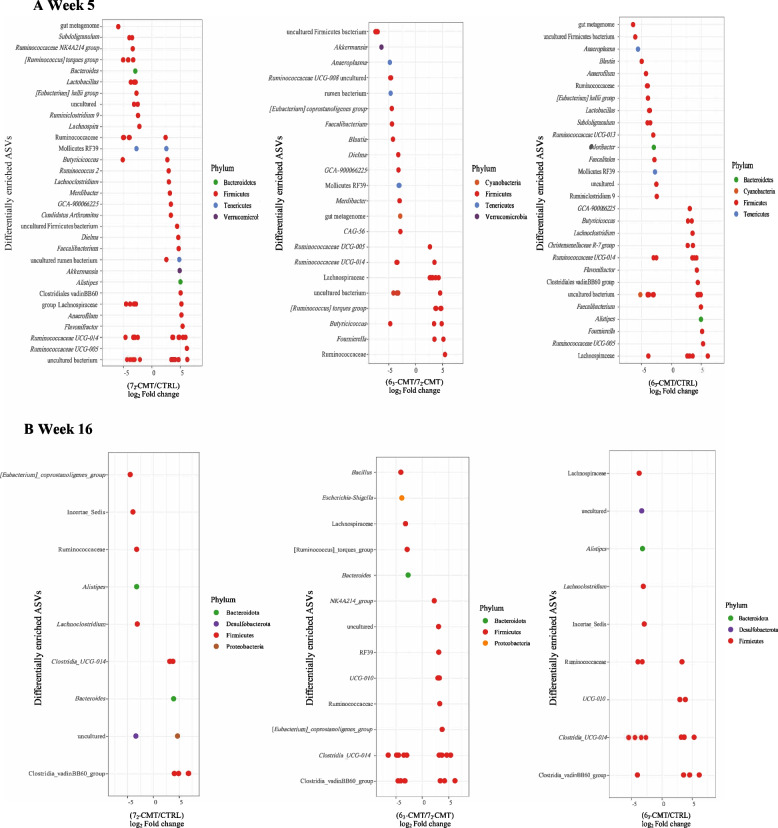
DESeq2 analysis of differentially abundant ASVs between 6_3_-CMT group and CTRL group, 6_3_-CMT group and 7_2_-CMT group, 7_2_-CMT group and CTRL group at **A** week 5 and **B** week 16. Estimations of log_2_ fold change values for each ASV were computed and each point represents an ASV that was significantly different (*P* ≤ 0.05). 6_3_-CMT, received cecal content solution from 6_3_ donors; 7_2_-CMT, received cecal content solution from 7_2_ donors; CTRL, received saline, control

The differentially abundant ASVs among the treatments were further examined (Fig. 4). At week 5, the number of differentially abundant ASVs was 38, 68, and 52, respectively, between the 6_3_-CMT chickens vs. 7_2_-CMT chickens, the 7_2_-CMT chickens vs. CTRL chickens, and the 6_3_-CMT chickens vs. CTRL chickens (Fig. 4A). Compared with the CTRL chickens, 9 ASVs belonging to *GCA-900066225, Lachnoclostridium, Christensenellaceae R7 group, Flavonifractor, Ruminococcaceae UCG-014, Faecalibacterium, Alistipes, Fournierella,* and *Ruminococcaceae UCG-005* were enriched in the 6_3_-CMT recipient chickens; while the 7_2_-CMT recipient chickens had 12 enriched ASVs belonging to *Alistipes, Candidatus* Arthromitus*, Dielma, Merdibacter, Faecalibacterium, Flavonifractor, GCA-900066225, Anaerofilum, Ruminococcus 2, Ruminococcaceae UCG-005, Ruminococcaceae NK4A214 group,* and *Lachnoclostridium*. Compared with the 6_3_-CMT recipient chickens, 10 ASVs belonging to *Akkermansia*, *Dielma*, *Merdibacter*, *Anaeroplasma*, *Ruminococcaceae UCG-008*, *Faecalibacterium*, *GCA-900066225*, *Ruminococcaceae UCG-014*, *CAG-56*, and *Blautia* were more abundant than those in the 7_2_-CMT recipient chickens.

At week 16, there were 30, 14, and 25 differentially abundant ASVs between the 6_3_-CMT chickens vs. 7_2_-CMT chickens, the 7_2_-CMT chickens vs. CTRL chickens, and the 6_3_-CMT chickens vs. CTRL chickens, respectively (Fig. 4B). Those ASVs belong to the phyla Bacteroidetes, Desulfobacterota, Firmicutes, and Proteobacteria. Compared with the CTRL chickens, 5 ASVs were more abundant (*Alistipes, Clostridia_UCG-014, Incertae_Sedis, Lachnoclostridium*, and Lachnospiraceae) in the 6_3_-CMT chickens, while the 7_2_-CMT chickens had 2 enriched ASVs belonging to genera *Bacteroides* and *Clostridia_UCG-014*. Compared with the 6_3_-CMT recipient chickens, 3 ASVs (*Bacillus*, *Escherichia-Shigella*, *Bacteroides*) were more abundant in the 7_2_-CMT chickens.

### Changes of brain monoamines in recipient chickens

**Fig. 5:**
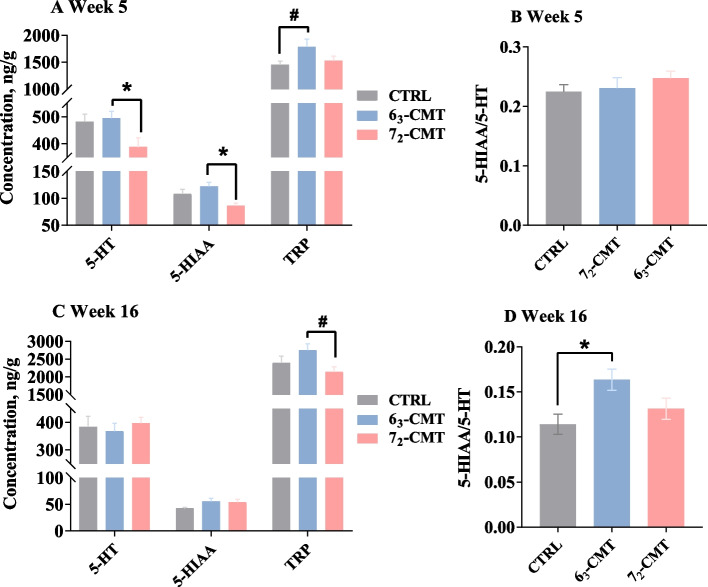
Effects of cecal microbiota transplantation on the parameters of the serotonergic activities in the hypothalamus of recipient chickens at **A**-**B** week 5 and **C**-**D** week 16. Values are least square means ± SEM, *n* = 7. ^*^Indicates significant differences (*P* ≤ 0.05), and ^#^shows trend differences (0.05 < *P* ≤ 0.1). 5-HIAA, 5-Hydroxuindoleacetic acid; 5-HT, serotonin; 6_3_-CMT, received cecal content solution from line 6_3_ donors; 7_2_-CMT, received cecal content solution from line 7_2_ donors; CTRL, received saline, control; TRP, tryptophan

**Fig. 6:**
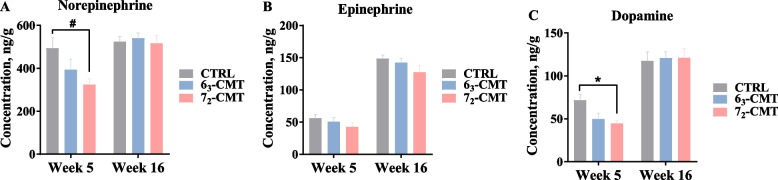
Effects of cecal microbiota transplantation on levels of **A** norepinephrine, **B** epinephrine, and **C** dopamine in the hypothalamus of recipient chickens at week 5 and week 16. Values are least square means ± SEM, *n* = 7. ^*^Indicates significant differences (*P* ≤ 0.05), and ^#^shows trend differences (0.05 < *P* ≤ 0.1). 6_3_-CMT, received cecal content solution from line 6_3_ donors; 7_2_-CMT, received cecal content solution from line 7_2_ donors; CTRL, received saline, control

Cecal microbiota transplantation-caused changes in the biomarkers of the central serotonergic system in recipient chickens are illustrated in Fig. 5. At week 5, the 6_3_-CMT chickens had higher concentrations of hypothalamic 5-HT (*P* = 0.037) and 5-HIAA (*P* = 0.007, Fig. 5A), while no differences were found for serotonin turnover (*P* > 0.05, Fig. 5B). Although there were no treatment effects on the levels of 5-HT and 5-HIAA at week 16 (Fig. 5C), 5-HT turnover in the 6_3_-CMT chickens was elevated compared to the CTRL chickens (*P* = 0.011, Fig. 5D). In addition, the 6_3_-CMT chickens tended to have higher concentrations of TRP in the hypothalamus compared with the CTRL chickens at week 5 (*P* = 0.089) and 7_2_-CMT chickens at week 16 (*P* = 0.063). Microbiota modulation also affected the activation of the noradrenergic system. At week 5, the 7_2_-CMT chickens had lower NE (*P* = 0.072, Fig. 6A) and DA concentrations (*P* = 0.031, Fig. 6C) compared to the CTRL chickens but not the 6_3_-CMT chickens. There were no treatment effects on NE, EP, and DA levels at week 16 (*P* > 0.05, Fig. 6). No differences were found for gene expressions of *TPH2*, *Htr1a*, *Htr1b*, and *5-HTT*, except that *MAOA* mRNA abundance was increased in the 6_3_-CMT group at week 16 (*P* = 0.024, Table 4).

**Table 4:** Effects of cecal microbiota transplantation on relative mRNA expression of genes involved in the serotonergic pathway in the hypothalamus of recipient chickens at week 5 and week 16

Treatment	*MAOA*	*TPH2*	*5-HTT*	*Htr1a*	*Htr1b*
Week 5					
6_3_-CMT	1.51	0.33	1.12	1.47	1.19
7_2_-CMT	1.56	0.45	0.72	1.44	1.45
Control	1.45	0.40	0.92	1.50	1.36
SEM	0.11	0.05	0.17	0.11	0.13
*P-*value	0.80	0.31	0.26	0.93	0.37
Week 16					
6_3_-CMT	2.52^a^	0.83	1.63	0.86	2.35
7_2_-CMT	1.80^b^	0.84	1.54	0.77	2.32
Control	1.90^ab^	1.00	2.04	0.81	2.12
SEM	0.05	0.12	0.21	0.07	0.09
*P-*value	**0.02**	0.45	0.23	0.67	0.18

^a,b^ Means with different superscript indicate significant difference (*P* ≤ 0.05). Values are least square means ± SEM, *n* = 7. *5-HTT* erotonin transporter, *6*_*3*_*-CMT* Received cecal content solution from 6_3_ donors, *7*_*2*_*-CMT* Received cecal content solution from 7_2_ donors, *CTRL* Received saline, control. *Htr1a* 5-HT 1a receptor, *Htr1b* 5-HT 1b receptor, *TPH2* Tryptophan hydroxylase 2, *MAOA* Monoamine oxidase A

### Alterations in plasma tryptophan and serotonin

**Fig. 7:**
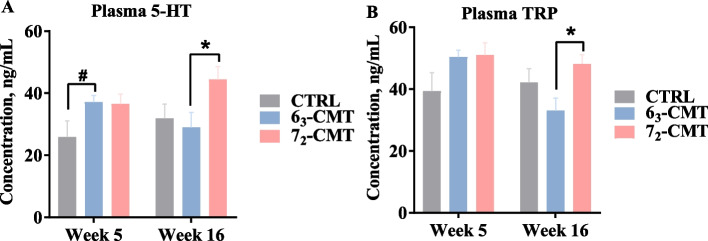
Effects of cecal microbiota transplantation on the plasma **A** 5-HT and **B** TRP concentration of recipient chickens at week 5 and week 16. Values are least square means ± SEM, *n* = 7. ^*^Indicates significant differences (*P* ≤ 0.05), and ^#^shows trend differences (0.05 < *P* ≤ 0.1). 5-HT, serotonin; 6_3_-CMT, received cecal content solution from line 6_3_ donors; 7_2_-CMT, received cecal content solution from line 7_2_ donors; CTRL, received saline, control; TRP, tryptophan

At week 5, the 6_3_-CMT chickens tended to have higher 5-HT concentrations in the plasma as compared to the CTRL chickens (*P* = 0.086, Fig. 7A), while no treatment effects on TRP levels (*P* > 0.05, Fig. 7B). At week 16, the 7_2_-CMT recipient chickens had higher levels of plasma 5-HT (*P* = 0.033) and TRP (*P* = 0.037) as compared to the 6_3_-CMT chickens.

### Correlations between neurotransmitter levels and differentially abundant ASVs

**Fig. 8:**
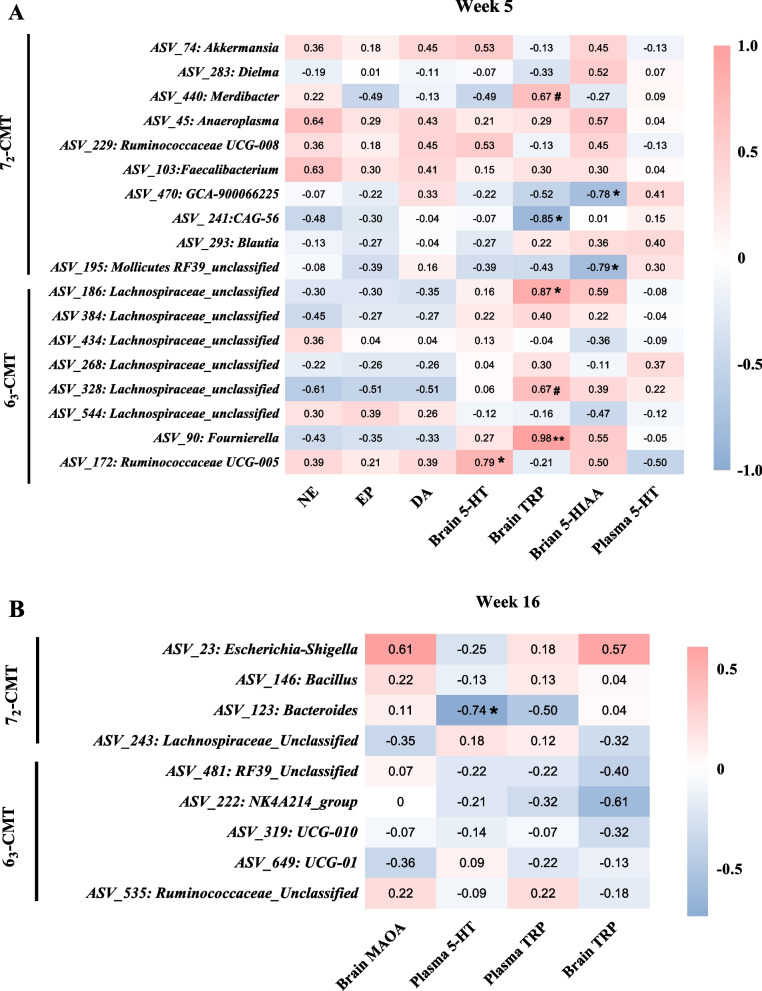
Spearman correlations of neurotransmitter (and its metabolites) concentration with bacterial taxa (ASVs) in 7_2_-CMT and 6_3_-CMT chickens at **A** week 5 and **B** week 16. ^**^Represents* P* < 0.001, ^*^indicates significant differences (*P* ≤ 0.05), and ^#^shows trend differences (0.05 < *P* ≤ 0.1). 5-HIAA, 5-Hydroxuindoleacetic acid; 5-HT, serotonin; 6_3_-CMT, received cecal content solution from line 6_3_ donors; 7_2_-CMT, received cecal content solution from line 7_2_ donors; CTRL, received saline, control; DA, dopamine; EP, epinephrine; MAOA, monoamine oxidase; NE, norepinephrine; TRP, tryptophan

At week 5, two ASVs belonging to Lachnospiraceae (*r* = 0.87 and 0.67, *P* = 0.012 and 0.100, Fig. 8A) and one *Fournierella* ASV (*r* = 0.98, *P* < 0.001) were positively correlated with the brain TRP in the 6_3_-CMT recipient chickens. In addition, there was a positive correlation between one *Ruminococcaceae UCG-005* ASV (*r* = 0.79, *P* = 0.036) and the brain 5-HT levels in the 6_3_-CMT chickens. No correlations were found in the 6_3_-CMT chickens at week 16 (Fig. 8B). In the 7_2_-CMT, one *Mollicutes RF39* ASV (*r* = −0.79, *P* = 0.035) and one *GCA-900066225* ASV (*r* = −0.78, *P* = 0.039) were negatively associated with the brain 5-HIAA, and one *CAG-56* ASV was negatively correlated with the brain TRP (*r* = −0.85,* P* = 0.015) at week 5, while at week 16, one *Bacteroides* ASV (*r* = −0.74, *P* = 0.048) was negatively correlated with the plasma 5-HT.

## Discussion

Growing evidence has highlighted the involvement of gut microbiota in the regulation of host physiological and behavioral homeostasis and the potential implications of gut microbiota for treating patients with psychiatric and mental disorders [43]. Here, we found transplantation of cecal content from nonaggressive and aggressive donors exerted different developmental patterns of the cecal microbial community, and the activities of central serotonergic and catecholaminergic systems, contributing to divergent behavior phenotypes in recipients, Dekalb XL.

Early-life gut microbiota development has long-term effects on health and disease in humans [44]. Similar outcomes have been revealed in chickens [45, 46]. In the present study, CMT at an early-age induced an increase in phylogenetic diversity in the 7_2_-CMT chickens (susceptible lines with greater aggressiveness) as compared with the 6_3_-CMT chickens (resistant lines with less aggressiveness) throughout the 16-week trial. Previous studies have shown that patients with neuropsychiatric disorders including Parkinson’s disease [47] and autism spectrum disorder [48] have a higher α-diversity as compared with healthy individuals, which may indicate the association between the gut microbial diversity and neurological disease exist. Similarly, in broiler chickens, *Eimeria* inoculation (a model for coccidiosis) induces an increase in cecal microbial richness and overall diversity compared to healthy control chickens [49]. In our study, the higher phylogenetic diversity in the 7_2_-CMT chickens may be associated with the susceptible properties to Marek’s disease found in the 7_2_ donors. Future studies will be needed to explore the underlying mechanisms regarding the diversity-disease relationship. In addition, the CMT-induced effects on the β-diversity were in an age-specific manner; that is, differences in cecal microbial structures were found at week 5 but not at week 16 as the recipients are close to sexual maturity (around 16–17 weeks of age). This may indicate that CMT had a greater influence on the establishment of gut microbiota at an early age, whereas at adulthood, host genotype, environmental factors, and genetic-environment interaction may have a more significant impact than CMT on the structure of the cecal microbial community in recipient chickens.

An ASV belonging to *Ruminococcaceae UCG-005* was abundant in the 6_3_-CMT chickens, a genus that has been reported to function as short-chain fatty acids (SCFAs) producers, by which it may indirectly influence the synthesis of neurotransmitters (e.g., 5-HT, TRP, or DA) and aggressive behavior [50, 51]. Similarly, different amounts of propionate and n-butyrate were found in ceca of hens with high and low repetitive feather-pecking behavior [52]. Also, propionic acid-treated rats exhibit different social behavior as compared with PBS-treated rats (regular controls) [53]. Correspondingly, several bacteria producing SCFAs have been used as psychobiotics [54] in treating psychiatric [55] and Alzheimer’s disease [56] via regulation of the microbiota-gut-brain axis. As such, it may indicate that gut microbiota (actual bacteria) functionally regulates mental health by influencing the serotonergic system. In support, we found that CMT from different donors affects levels of brain neurotransmitters in recipient chickens. Compared to 7_2_-CMT chickens, 6_3_-CMT chickens had higher concentrations of 5-HT at week 5 and increased 5-HT turnover rates and TRP levels in the hypothalamus at week 16. In humans, blocking 5-HT transporters has been used as an efficient therapy for patients with social impulsivity or aggressiveness [57]. Dennis et al. [58] also reported that excessive embryonic 5-HT administration modulates aggressive and fearful behaviors during sexual maturity in chickens. Similar to our findings, the elevated hypothalamic 5-HT levels supported the behavioral observations from both home cages and paired tests: 6_3_-CMT chickens had higher hypothalamic 5-HT concentrations with less aggressive pecking as compared with 7_2_-CMT chickens. Moreover, 5-HIAA, as the metabolite of 5-HT, is thought to be negatively correlated with aggression [59]. Interestingly, 6_3_-CMT chickens exhibited higher levels of 5-HIAA among the treatment groups, which may indicate elevated serotonergic activities (higher 5-HIAA/5-HT ratio). As discussed before, the SCFA-producer genus *Ruminococcaceae UCG-005* may indirectly enhance the 5-HT levels by influencing the TRP synthesis in the gut. In support, we also found strong positive correlations between the ASVs belonging to *Fournierella* and *Lachnospiraceae* with the brain TRP in the 6_3_-CMT chickens. Tryptophan, as a precursor of 5-HT, can be regulated by certain intestinal bacterial strains and is involved in the synthesis of central 5-HT. Thus, it is likely that *Fournierella* and *Lachnospiraceae* may indirectly influence 5-HT levels by affecting tryptophanase enzyme activities or indole production capacity [60]. Additionally, we found that 7_2_-CMT chickens had enriched ASVs belonging to *Bacillus*, *Escherichia*, and *Bacteroides* compared to the 6_3_-CMT chickens at week 16. These genera have been recognized as opportunistic pathogens producing exotoxins to accelerate inflammation in the hosts [61]. In chickens, enriched *Escherichia* may disrupt the colonization of resident bacteria, thereby increasing the susceptibility to other pathogens [49]. Exposure to pathogenic bacteria in the gut induces behavioral problems in humans including depression-like behavior [62]. Goehler et al. [63] also found that patients with cognitive issues exhibited overgrowth of *Escherichia* in the gut, which is similar to our findings that 7_2_-CMT chickens tended to exhibit more aggression during paired tests. Collectively, gut bacteria may influence aggressive behavior in recipient chickens by enhancing hypothalamic serotonergic neurotransmission.

Generally, the activity of the 5-HTergic system is regulated by the enzyme tryptophan hydroxylase (TPH), a rate-limiting enzyme of 5-HT synthesis, and monoamine oxidase (MAO), an enzyme that catalyzes monoamine production including 5-HT [64]. Although we did not observe the CMT-induced changes in the abundance of *TPH2* mRNA in the brain, a significantly higher expression of brain *MAOA* gene was found in the 6_3_-CMT chickens at week 16, which may correlate with a tendency for less aggression. It has been proposed that the catalytic activity of *MAOA* gene in the brain may serve as a biomarker for assessing aggression [65]. In a clinical study, individuals with antisocial personality disorders have lower levels of brain MAOA compared with controls, suggesting the inversive correlation between MAOA levels and aggression [66]. In general, the up-regulation of brain MAOA is associated with enhanced 5-HT turnover, pointing to lower aggressive behavior [67]. Interestingly, 6_3_-CMT chickens had higher concentrations of TRP and 5-HT turnover ratios, which may indicate gut microbiota regulates aggressive behavior via alterations in the serotonin metabolism. It is worth to note that the identified serotonergic pathway has also been previously reported to be involved in damaging feather pecking [68–70].

It has been reported that genus *Akkermansia* facilitates the degradation of the mucin layer and increases intestinal permeability, by which it may have a prominent role in the early development of neurodegenerative diseases such as Parkinson’s disease [71]. McGaughey et al. [72] have reported the abundance of *Akkermansia* spp. was positively correlated with the behavioral metrics of both anxiety and depression. Additionally, previous studies found a positive correlation between the abundance of an *Akkermansia* spp. and tyrosine levels in cecal contents of layer-type pullets vaccinated with live *Salmonella* [73]. Increased tyrosine may influence NE levels, thereby affecting brain function and behavioral pattern. Norepinephrine, as a neuromodulator, regulates the activity of both neuronal and nonneuronal cells via multiple pathways that participated in the neuroplasticity during growth, stress response, and inflammatory process [74, 75]. Though NE cannot penetrate the blood–brain barrier to enter the brain, as an essential amino acid and a precursor of NE, tyrosine could be transported to brain neurons and used for synthesizing catecholamines to influence brain function [76]. In the current study, 7_2_-CMT chickens showing more aggressive pecking had the lowest hypothalamic NE among the treatments at week 5. An early study in rodents showed that stress-induced increases in NE could be attenuated by the exhibition of aggression via negative feedback of noradrenergic system [77]. Likely, the enriched *Akkermansia* ASV in the 7_2_-CMT recipient chickens may play a similar role in regulating aggression at an early age (at week 5). The CMT-altered dopaminergic transmission was further observed among the recipient chickens, with the lowest concentrations of hypothalamic DA in the 7_2_-CMT chickens at week 5. Though the role of dopamine in the regulation of aggression remains unspecified, Schlüter et al. [78] reported a negative correlation between aggressive actions and the subcortical (mainly the midbrain) DA-synthesis capacity. Dopamine is also involved in coping with stress in risk-taking via the reward system [79] and the mesocorticolimbic dopamine system [80]. Patients with Parkinson's disease have lower levels of DA in the nigrostriatal pathways with a great number of risky decisions [81]. Taken together, the reduced DA in the 7_2_-CMT chickens further support our hypothesis that gut microbiota may work together with the catecholaminergic system to modulate aggression.

## Conclusion

The current results indicate that CMT at an early age affects the development of aggressive behavior in recipient chickens via regulating the activities of brain serotonergic and catecholaminergic systems. The outcomes provide new insights into understanding the mechanism of aggression in poultry through the microbiota-gut-brain axis. These results suggest a potential strategy of early intervention with the gut microbiota to reduce aggressive behavior and improve welfare for poultry production.

## Data Availability

The datasets used and analyzed in the current study are available from corresponding authors on reasonable request. All data generated and analyzed during this study are included in this published article.

## Abbreviations

5-HIAA: 5-Hydroxuindoleacetic acid
5-HT: Serotonin
5-HTergic: Serotonergic
5-HTT: Serotonin transporter
6_3_-CMT: Chickens with cecal solution of line 6_3_
7_2_-CMT: Chickens with cecal solution of line 7_2_
ASVs: Amplicon sequence variants
BW: Body weight
CMT: Cecal microbiota transplantation
CNS: Central nervous system
CTRL: Chickens with saline, control
DA: Dopamine
ELISA: Enzyme-linked immunosorbent assay
EP: Epinephrine
FMT: Fecal microbiota transplantation
GF: Germ-free
HPLC: High-performance liquid chromatography
Htr1a: 5-HT 1a receptor
Htr1b: 5-HT 1b receptor
MAO: Monoamine oxidase
NE: Norepinephrine
SCFAs: Short-chain fatty acids
SPF: Specific pathogen-free
TPH: Tryptophan hydroxylase
TRP: Tryptophan
